# A Case of Obstruction of the Colon, Relieved by an Operation Performed at the Groin

**Published:** 1851-10

**Authors:** James Luke


*A Case of Obstruction of the Colon, relieved by an Operation performed at the Groin.* By James Luke, Esq.—The subject of this report was a man aged sixty, who, on December 16, 1850, first complained to the author of feeling generally unwell. He had no pain, but his countenance was depressed, his eyes sallow, and his tongue coated. The bowels were confined, and lately medicine had acted with difficulty on them. An aperient was ordered, and on the following day he passed a small lumpy motion, but without relief to the symptoms; castor-oil was ordered, but after a time was rejected by vomiting. On the 18th, there was no relief from the bowels; and he vomited everything he took. From this time, he progressively got worse, in spite of all the means resorted to for his relief. He complained of pain chiefly about the region of the cæcum. The transverse arch of the colon could be felt distended and tympanitic. A careful observation of the case had led the author to believe, that there was obstruction in the bowel about the sigmoid flexure of the colon, and it was resolved, as a last resource, to operate. The operation was performed on the 23rd. Mr. Luke opened the abdominal parietes near the groin, by an incision four inches in length, a little to the outside of the course of the epigastric artery, the lower extremity of which incision terminated a little above Poupart's ligament. The peritoneum was opened to about two inches. On passing the finger down the surface of the intestine, which now protruded, a diseased mass could be felt, which appeared to encircle the intestines. The bowel was then opened above this part; a large quantity of fæculent matter came away, and the patient expressed himself as relieved. On now passing the finger into the bowel, it was found to be impervious about two inches below the aperture. After the operation, the recovery of the patient was rapid. On the second day, fæces passed *per anum*, and continued to do so for more than a month, when their passage through the natural opening ceased; it was again partially restored, but from this time the greater part of the fæces passed by the wound. This was closed by a well-fitted pad, and he had been enabled since to pursue his ordinary occupation almost without interruption.

The author then remarked on the danger of protracted delay in relieving such cases; a delay which is, however, to a great extent rendered necessary by the difficulties of diagnosis. The distension of the colon and the evidence afforded by the proper introduction of the long tube, were pointed out as the two most accurate means for determining the seat of obstruction, when it is situated at the lower part of the colon. The advantages of the operations of Amussat and Littre were then compared, and the author, while admitting the advantage gained by operating in the loins, as proposed by the former—of not opening the peritoneal cavity—yet thought that the operation in the groin offered certain advantages. By the operation in the loins nothing more could be done than opening the intestine; but this might in some cases be improper—as where obstructions were produced by fibrous bands overlaying the intestine, or by strangulations, the result of causes acting exteriorly to its tunics. In these cases, the proper treatment is to divide the bands, or relieve the cause of strangulation. In the event, too, of an error of diagnosis, the opening in the loins does not provide any facilities for correcting the error. The danger of total failure of affording relief consequent upon this state of things, must therefore be attributable as a demerit to the operation in the loins. Besides, the opening cannot be conveniently attended to by the patient himself, and there exists frequently a great disposition to contraction, from the great depth of the wound, which requires renewed surgical interference. With the exception of the necessary peritoneal section, the operation of opening the abdominal parietes at the groin, in all cases of obstruction, or suspected obstruction, in the lower part of the colon, appeared to the author to be preferable. It affords facilities for modifying the treatment, either by opening the intestine, when incapable of relief by other means, or by dividing or removing any existing cause of strangulation. It enables the surgeon to extend his search within a limited range, in the event of the diagnosis proving incorrect; it allows him to open the bowel as close as possible to the seat of obstruction, and it secures to the patient the facilities for attending to his own comfort.—*Lon. Jour. of Med.*

